# Rifampicin-Induced Pulmonary Embolism: A Rare Side Effect

**DOI:** 10.7759/cureus.18904

**Published:** 2021-10-19

**Authors:** Mouhammad J Alawad, Mhd Kutaiba Albuni, Eihab A Subahi, Ijaz Kamal

**Affiliations:** 1 Internal Medicine Residency Program, Medical Education, Hamad Medical Corporation, Doha, QAT; 2 Internal Medicine, Hamad Medical Corporation, Doha, QAT

**Keywords:** rifampicin, tuberculosis, pulmonary embolism, deep vein thrombosis, venous thromboembolism

## Abstract

Rifampicin is an established and effective antibiotic and a gold standard in treating tuberculosis (TB). Venous thromboembolism (VTE) events are a rare side effect of rifampicin, which has been reported in a few case reports. The exact mechanism is yet not clear, however, could include immunological and hematological causes. Here, we report a 56-year-old male who presented with pulmonary embolism (PE) three weeks after initiating rifampicin for latent TB management. Comprehensive investigations were done to rule out any other causes of thrombosis, especially malignancy, however, all tests were negative. The patient was treated with anticoagulant agents and rifampicin was switched to isoniazid after rifampicin discontinuation. He remained stable upon discharge and follow-up.

## Introduction

Rifampicin is an RNA polymerase inhibitor class antibiotic, and it is still one of the most powerful antibiotics since its discovery in the 20th century. Its use is reserved for serious bacterial infections, such as active and latent tuberculosis (TB) and leprosy [1]. Rifampicin is associated with many adverse reactions, side effects, and drugs interactions, the majority of which have been studied and described in the literature. However, venous thromboembolism (VTE) events, such as deep vein thrombosis (DVT) and disseminated intravascular coagulation (DIC), have been postulated and linked to rifampicin in a few cases reports. However, the exact mechanism is not fully understood, but a multifactorial process is likely playing a role [2,3]. An unprovoked VTE should be approached carefully, as this entity is quite a common complication to numerous medical illnesses. Herein, we present a patient with celiac disease and latent TB that developed DVT with a massive saddle pulmonary embolism (PE), three weeks after starting rifampicin.

## Case presentation

A 56-year-old male, with a past medical history of diabetes mellitus type 2, hypothyroidism, and celiac disease. He had a history of reactive abdominal and left axillary lymphadenopathy, which was diagnosed by imaging 18 months ago and was stable after repeated scans. He was diagnosed incidentally with latent tuberculosis with a positive QuantiFERON test and was started on rifampicin. Three weeks after starting rifampicin, the patient presented to the emergency department with chest pain. It was centrally located, sudden in onset, squeezing in nature, and with no radiation. The pain lasted for nearly 20 minutes. It was not associated with shortness of breath, hemoptysis, or cough. The patient mentioned that he had a weight loss of around 5 kg over the last couple of months. He had no history of immobility, travel history, or surgical interventions. 

**Figure 1:**
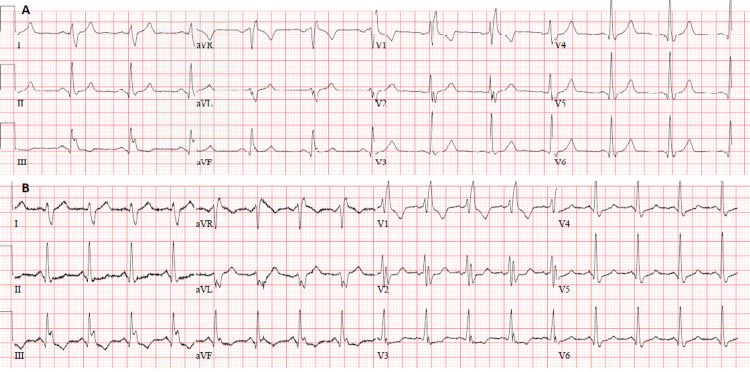
Electrocardiogram (A) ECG of the patient six months prior to presentation and (B) ECG on day of presentation.

**Figure 2:**
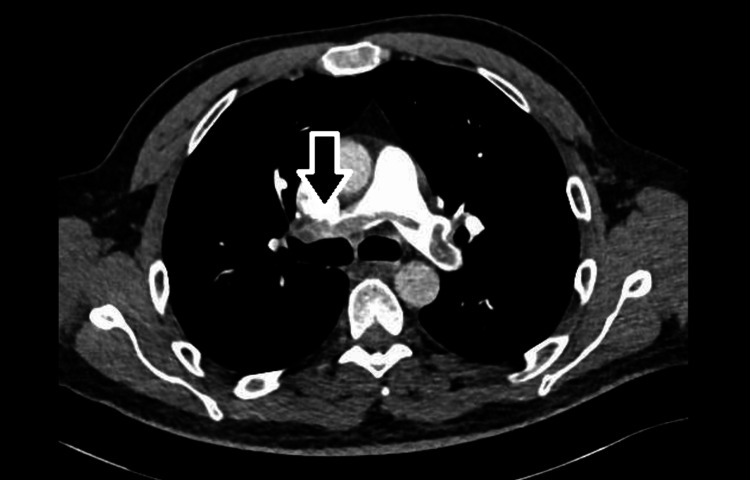
CT pulmonary angiography CT pulmonary angiography protocol, at the bifurcation of pulmonary trunk showing the filling defect (arrow).

The patient was not in distress. Vitally: temperature 37.1 °C, blood pressure (BP) 119/67 mmHg, heart rate (HR) 91 beat per minute, saturation 96% on room air, body mass index (BMI) 27.2. Physical examination was remarkable for mild hepatomegaly, multiple, small, and enlarged lymph nodes in the axillary and inguinal area, with being the largest in the left axilla measuring 1.5 cm. Lower limbs examination revealed stiffness in the left calf but no asymmetry, redness, or tenderness. An electrocardiogram (ECG) was done, showing new changes (T-wave inversion in III, aVF, V3, mild ST-segment depression in V3-V6), along with an old right bundle branch block (Figure 1). Chest X-ray showed no obvious pulmonary infiltrates/consolidation, the costophrenic angles were clear, and no apparent cardiomegaly. The patient’s D-Dimer has elevated at 8.01 mg/L FEU and positive troponin at 235 ng/L (normal range max approximately 13 ng/L). All other hematological and biochemical results were within the normal range. Given the clinical scenario with ECG and lab results, CT pulmonary angiography was done, indicating a filling defect seen in bilateral pulmonary arteries extending into the main segmental branches and draped over the bifurcation of the main pulmonary artery, consistent with saddle PE (Figure 2). The patient was started on enoxaparin 80 mg BID. Ultrasound Doppler of lower limbs confirmed evidence of DVT, noted at the left, distal saphenofemoral vein (SFV), and popliteal vein levels, showing partial thrombus in the lumen with the partial flow. Extensive thrombophilia and autoimmune workup perturbing antinuclear antibody, antineutrophil cytoplasmic autoantibody, rheumatoid factor, C3, C4, homocysteine levels, factor V Leiden, lupus anticoagulant, protein C, protein S, antithrombin activity, anticardiolipin, IgM and IgG, and anti-B2 glycoprotein IgG, were all negative. Thyroid-stimulating hormone (TSH) was 2.55 mIU/L (normal range: 0.30-4.20) and free T4 of 15.1 pmol/L (normal range: 11-23).

Due to the history of weight loss and lymphadenopathy, an underlying malignancy was of concern; therefore, a repeat PAN CT scan was done. This showed mild hepatosplenomegaly with mildly enlarged upper abdominal lymph nodes and left axillary lymph nodes, which are unchanged since June 2020. An ultrasound-guided core needle left axillary lymph node biopsy was organized and done. The biopsy results showed reactive lymphoid follicles and an unremarkable inter-follicular zone. The biopsy was negative for TB and mycobacterial cultures were negative after 42 days. The patient also underwent a whole-body positron emission tomography (PET) CT, which showed reactive left axillary lymph nodes. No other hypermetabolic lymph nodes were seen. There was no sign of lymphoma or any other fluorodeoxyglucose (FDG)-avid malignancy on PET CT.

After discussion with infectious disease specialists and hematologists, the patient was started on isoniazid and pyridoxine for latent TB. The patient was already on anticoagulation with dabigatran after ruling out malignancy. The patient will be closely followed up in the infectious disease clinic due to the side effects and drug interactions of his current regimen.

## Discussion

Venous thromboembolism events are classified as provoked and unprovoked, based on preceding circumstances, such as trauma, surgery, or prolonged immobilization, which is important for the management and duration of treatment [4]. Our assessment is that this episode of VTE was triggered by the use of rifampicin, which was started three weeks prior to the presentation. Rifampicin was linked in a few case reports to coagulation disorders, leading to VTE or DIC [2,3,5]. Moreover, the exact mechanism is thought to be related to the metabolism of procoagulant and anticoagulant proteins [2,3]. However, an immunologic process has also been described [3]. Statistically, rifampicin has an increased relative risk for DVT [2,3]. The discontinuation of rifampicin was associated with regression of DVT, as reported by Sarkar et al. [5]. The development of rifampicin-related coagulopathy can happen anywhere and anytime, from hours to six months after starting rifampicin [6,7].

An underlying malignancy was a big concern because of the patient’s age and history of weight loss with associated findings of lymphadenopathy. However, PET CT scan and lymph node biopsy were both negative for malignancy and suggestive of reactive changes. A follow-up repeat PAN CT scan of chest abdomen showed regression in size of bilateral lobar and segmental pulmonary emboli, with a resolution of the previous main pulmonary arteries’ extension after withdrawal of rifampicin. Additionally, all autoimmune and thrombophilia workups were negative; however, this patient needs very close follow-up.

Tuberculosis is also associated with some cases of VTE [8,9]. A multifactorial process is often implicated like chronic inflammation, hypercoagulable state, or direct endothelial injury [8,9]. However, it is worth noting that the reported cases were of active TB.

Celiac disease has also been reported as a risk factor for VTE [10]. Usually, the risk of VTE is highest within the first 90 days to one year after the diagnosis of celiac disease, with a possible reduction in risk by following a gluten-free diet [10]. Celiac disease has been diagnosed in our patient four years ago, and he adheres well to dietary restrictions, as per celiac diet recommendations.

## Conclusions

Special attention should be paid when starting a rifampicin-based anti-tubercular medication. Rifampicin is the likely cause of VTE in our patients. However, it remains a rare cause of VTE and every possible effort should be made to rule out other potential causes of VTE like malignancy, infection, or autoimmune. A detailed history and physical examination need to be carried out and in few cases an extensive workup too. An individualized approach is recommended. VTE requires early recognition and proper management. A close follow-up is required for such patients in a dedicated anticoagulation clinic.
